# Use of adjunctive glycaemic agents with vascular protective properties in individuals with type 1 diabetes: Potential benefits and risks

**DOI:** 10.1111/dom.16332

**Published:** 2025-03-25

**Authors:** Ahmad M. Rajab, Sam Pearson, Ramzi A. Ajjan

**Affiliations:** ^1^ Diabetes Centre St James's University Hospital, Leeds Teaching Hospitals Trust Leeds UK; ^2^ Leeds Institute of Cardiovascular and Metabolic Medicine, Faculty of Medicine and Health University of Leeds Leeds UK

**Keywords:** diabetes complications, GLP‐1 analogue, insulin resistance, metformin, SGLT2 inhibitor, type 1 diabetes

## Abstract

Glycaemic therapy in type 1 diabetes (T1D) is focused on insulin, with the majority of studies investigating different insulin preparations, delivery devices and dosing accuracy methods. While insulin deficiency is the key mechanism for hyperglycaemia in T1D, individuals with this condition can also develop insulin resistance (IR), making optimisation of glycaemia more challenging. Importantly, IR in T1D increases the risk of both microvascular and macrovascular complications; yet, it is rarely targeted in routine clinical care. In this narrative review, we briefly discuss the mechanistic pathways for diabetes complications in individuals with T1D, emphasising the adverse role of IR. We subsequently cover the use of adjunctive glycaemic therapies for improving the metabolic profile in T1D, focusing on therapies that have possible or definite cardiovascular or renal protective properties in individuals with type 2 diabetes. These include metformin and agents in the thiazolidinedione, Sodium‐Glucose Cotransporter‐2 inhibitor (SGLT2i) and Glucagon‐Like Peptide‐1 Receptor Agonists (GLP‐1RA) groups. In addition to reviewing the role of these agents in improving metabolic parameters, we address their potential vascular and renal protective effects in individuals with T1D. We suggest a pragmatic approach for using these agents in T1D, based on current knowledge of their benefits and risks, while also highlighting gaps in knowledge and areas that require further research. It is hoped that the review raises awareness of the role of adjunctive therapies in T1D and offers healthcare professionals simple guidance on using such agents for the management of high‐risk individuals with T1D.

## 1 INTRODUCTION

The incidence and prevalence of T1D are on the rise, with the condition characterised by an immune‐mediated destruction of the insulin‐producing pancreatic β‐cells, thus necessitating exogenous insulin treatment.^1^ In 2021, there were around 8.4 million individuals worldwide living with T1D,^2^ a number that is expected to increase to between 13.5 and 17.4 million by 2040. Life expectancy varies considerably according to the care received, and for a 10‐year‐old diagnosed with T1D, subsequent years lived range from 13 years in low‐income countries to 65 years in high‐income countries.^2^ Overall, therefore, individuals with T1D have a higher risk of premature mortality compared to the general population^3^, ^4^, ^5^ due to acute complications such as diabetic ketoacidosis, hypoglycaemia and chronic vascular complications with longer duration of the condition.^6^, ^7^, ^8^ Early optimisation of glycaemia in T1D is crucial for reducing and delaying the incidence of both microvascular and macrovascular complications, as evidenced by the landmark diabetes control and complications trial (DCCT)^9^ and its extension, Epidemiology of Diabetes Interventions and Complications (EDIC),^10^ as well as many other studies.^11^, ^12^

Replacing insulin in T1D is the main management modality to control glucose levels through multiple daily insulin injections, continuous subcutaneous insulin infusion, and more recently, the use of a hybrid closed‐loop insulin delivery system.^13^, ^14^ However, it is not only glycaemia that is implicated in T1D complications, as insulin resistance (IR) also plays a role (detailed below). Data from the Pittsburgh Epidemiology of Diabetes Complications (EDC) study, which tracked participants for 18 years, showed a 47% increase in overweight T1D individuals, while the prevalence of obesity rose sevenfold, well above the rate observed in the general population.^15^ Therefore, the management of T1D is not all about glycaemia, and consideration should be given to tackling IR, which has yet to make it into routine clinical practice.^16^, ^17^

The main aim of this narrative review is to address the role of adjunctive therapies in reducing IR and vascular complications in T1D. We focus on agents that have shown possible or definite cardiovascular and/or renal protective properties in large‐scale type 2 diabetes studies.

## 2 MECHANISTIC PATHWAYS FOR THE VASCULAR COMPLICATIONS IN TYPE 1 DIABETES

The micro‐ and macrovascular complications of T1D not only jeopardise the quality of life and reduce the lifespan of patients with diabetes but also impose an economic burden on healthcare systems worldwide. It is important to understand that these complications arise not only from hyperglycaemia but also from hypoglycaemia and glycaemic variability, along with a complex interplay with IR and inflammatory pathways. Genetic factors are also likely to be influential.

To optimise diabetes management and mitigate vascular complications in T1D, it is critical to understand the distinct impacts of hyperglycaemia, hypoglycaemia and glycaemic variability. Each of these factors plays a unique role in the pathogenesis of vascular complications and may be differentially influenced by adjunctive therapies.

### 2.1 Glycaemic metrics and vascular complications in type 1 diabetes

Several mechanisms have been proposed to explain how chronic hyperglycaemia leads to vascular complications in T1D.^18^ The accumulation of Advanced Glycation End‐products (AGEs) as a result of hyperglycaemia can impair the function of proteins critical for vascular health while also inducing oxidative stress and inflammation.^19^ The polyol pathway is activated under hyperglycaemic conditions, where excess glucose is converted to sorbitol by aldose reductase, which contributes to oxidative stress. This pathway also consumes NADPH, reducing its availability for regenerating glutathione, a critical antioxidant.^20^

Oxidative stress damages endothelial cells, impairs nitric oxide production (essential for vascular dilation), induces mitochondrial dysfunction, and increases the production of proinflammatory cytokines, thus promoting atherogenesis. Hyperglycaemia and oxidative stress also activate protein kinase C (PKC), a family of enzymes that regulate various cellular functions, including blood flow, vascular permeability and the expression of pro‐inflammatory molecules.^21^

Treatment with insulin is associated with an increased risk of hypoglycaemia, which triggers a cascade of physiological responses that exacerbate vascular risk. These include activation of inflammatory and thrombotic pathways, which contribute to adverse vascular outcomes.^22^, ^23^, ^24^ This explains the recent international guidelines advocating hypoglycaemia avoidance in diabetes, particularly in those at high vascular risk.^25^

In addition to hyper‐d hypoglycaemia, fluctuation in glucose levels, referred to as glycaemic variability (GV), has been identified as an additional vascular risk factor through promoting oxidative stress and creating an inflammatory/thrombotic environment.^26^, ^27^

### 2.2 Insulin resistance and vascular complications in type 1 diabetes

Although IR is traditionally associated with T2D, it is increasingly recognised as a relevant factor in T1D. IR in T1D can be a consequence of lifestyle choices but may also be related to the management of the condition itself.^28^ IR is marked by a diminished tissue response to insulin, which exacerbates hyperglycaemia through unsuppressed hepatic gluconeogenesis and decreased muscular glucose uptake, requiring intensification of exogenous insulin therapy.^29^ Prolonged high insulin levels can cause internalisation and degradation of insulin receptors, necessitating even higher insulin doses to achieve the same glucose‐regulating effect.^30^ Hyperinsulinaemia, in turn, can lead to weight gain, further perpetuating the cycle of hyperinsulinaemia, obesity, escalating insulin requirements and worsening IR.^31^ Higher insulin doses increase the risk of hypoglycaemia, which can lead to maladaptive eating behaviours that contribute to the onset of obesity.^32^ It should be noted that subcutaneous administration of insulin, rather than the physiological delivery into the portal vein, predisposes to peripheral insulin resistance, constituting yet another mechanism for IR in T1D. The combination of glycaemic abnormalities, described above, together with increasing IR in T1D, increases the risk of cardiovascular complications by promoting atherogenic dyslipidaemia, hypertension and a pro‐inflammatory state.^33^, ^34^, ^35^, ^36^ The elevated levels of inflammatory cytokines (e.g., TNF‐α, IL‐6) in IR increase vascular permeability, attract immune cells to the endothelium, and exacerbate vascular damage.^37^ IR causes endothelial cell dysfunction, thus increasing production of the vasoconstrictor endothelin‐1 and reducing production and action of the vasodilator and anti‐thrombotic nitric oxide,^38^, ^39^ with the next results of increased peripheral vascular resistance and promotion of a prothrombotic environment.^31^, ^40^

**FIGURE 1:**
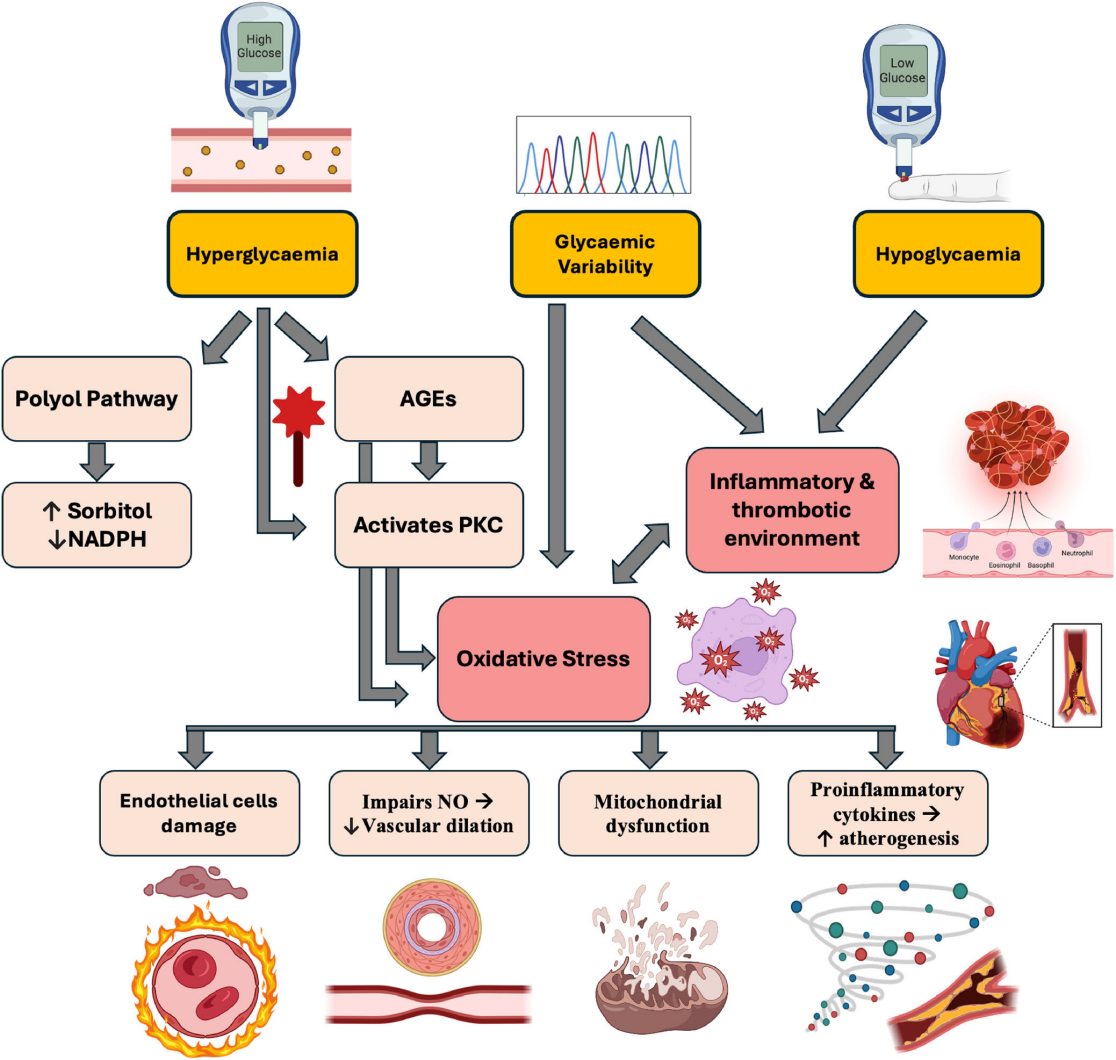
Mechanistic Pathways Leading to Vascular Complications in Type 1 Diabetes. This diagram illustrates the biochemical and physiological pathways activated by hyperglycaemia, hypoglycaemia and glycaemic variability in type 1 diabetes (T1D). Key processes include the activation of the polyol pathway, leading to oxidative stress via increased sorbitol and decreased Nicotinamide adenine dinucleotide phosphate (NADPH), the formation of Advanced Glycation End‐products (AGEs) which activate protein kinase C (PKC), and the resultant oxidative stress contributing to endothelial damage, impaired nitric oxide (NO) production and mitochondrial dysfunction. These changes culminate in increased proinflammatory cytokine activity, enhanced atherogenesis and increased risk of intra‐vascular thrombosis.

**FIGURE 2:**
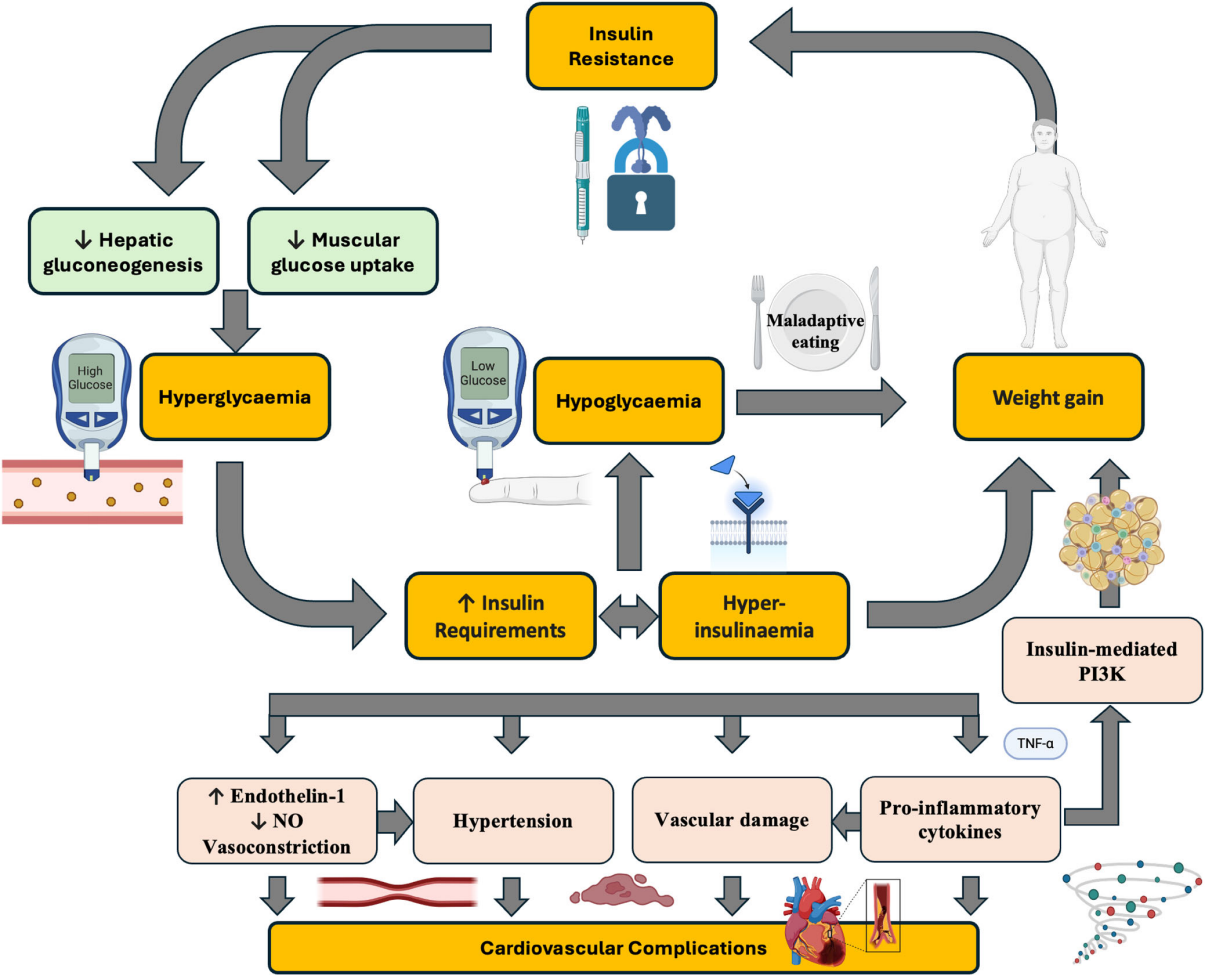
Interplay of Insulin Resistance, Hyperglycaemia and Vascular Outcomes in Type 1 Diabetes. This diagram highlights the role of insulin resistance (IR) in exacerbating hyperglycaemia and vascular complications in T1D. IR leads to increased hepatic gluconeogenesis and decreased glucose uptake by muscles, with the net result of higher blood glucose levels, necessitating increases in insulin doses. In turn, chronic hyperinsulinaemia promotes weight gain, further insulin resistance and a cascade of metabolic dysfunctions, including increased endothelin‐1 levels and decreased NO availability, leading to further vascular damage. These interconnected pathways illustrate the complex interplay of metabolic disturbances in T1D and their contribution to cardiovascular complications.

The combination of T1D and IR has led to the loosely defined subgroup of individuals with double diabetes (DD) who are at higher risk of both microvascular and macrovascular complications as detailed elsewhere.^28^ Despite accumulating evidence showing an adverse role for IR in T1D, routine practice remains focused on “target glucose levels” managed solely with insulin preparations, which can exacerbate IR.^31^ Therefore, more effective management strategies are required to improve insulin sensitivity in T1D, normalise the metabolic environment and reduce vascular risk (Figures 1 and 2).

### 2.3 Endothelial dysfunction in diabetes and the role of adjunctive therapy

Beyond oxidative stress and inflammation, additional mechanisms involving nitric oxide (NO) and the endothelial glycocalyx can lead to endothelial dysfunction, which is a major culprit in diabetes‐related vascular complications. Adjunctive therapies such as SGLT2i and GLP‐1RA can significantly enhance NO bioavailability by upregulating endothelial nitric oxide synthase (eNOS) thus improving endothelial function.^41^, ^42^ The endothelial glycocalyx is a critical vascular barrier, and its enhanced degradation by hyperglycaemia predisposes one to cardiovascular disease. However, therapies like SGLT2i and GLP‐1RA have been shown to support the structural integrity of the glycocalyx, offering protection against atherogenic changes.^43^ Moreover, agents such as metformin, empagliflozin and semaglutide enhance circulating endothelial cells (CECs) and endothelial progenitor cells (EPCs), consequently improving vascular regenerative potential.^44^, ^45^

## 3 ADJUNCTIVE THERAPIES TO IMPROVE VASCULAR OUTCOMES IN T1D

Given the adverse vascular effects of IR, new management strategies should be adopted to address this increasingly prevalent metabolic abnormality in T1D.^46^, ^47^, ^48^ We discuss here the potential benefits and risks of four adjunctive therapies in T1D that can modulate IR and are believed to offer cardiovascular protection in individuals with T2D. These agents are metformin, pioglitazone, sodium‐glucose cotransporter‐2 inhibitors (SGLT2i) and glucagon‐like peptide‐1 receptor agonists (GLP‐1RA). It should be noted that randomised controlled trials showing cardiovascular benefits in T2D exist for only three classes (thiazolidinedione, SGLT2i and GLP‐1RA),^49^, ^50^, ^51^ while for metformin, some observational studies suggest cardiovascular benefits^52^ but this remains debatable.

## 4 METFORMIN

Metformin is believed to improve insulin sensitivity in T2D, reducing hepatic glucose production and increasing glucose uptake by skeletal muscles and adipocytes.^53^ Given these actions, metformin has been proposed to lower insulin requirements and mitigate weight gain in individuals with T1D.^54^

### 4.1 Clinical benefits of metformin in T1D

To date, relatively few studies have explored the efficacy and safety of combining metformin with insulin in T1D patients.^54^, ^55^, ^56^, ^57^, ^58^ An early meta‐analysis of short‐term heterogeneous studies concluded that the addition of metformin may reduce both insulin requirements and weight in individuals with T1D, though it was unclear whether this was sustained beyond 1 year of treatment. Notably, there was no significant reduction in HbA1c and none of the studies investigated cardiovascular outcomes.^59^ Building on these findings, a systematic review and meta‐analysis has shown that metformin improves glycaemia and lipid profile, reduces diastolic blood pressure, prevents weight gain and reduces carotid artery intima‐media thickness, without significantly increasing diabetic ketoacidosis, lactic acidosis or hypoglycaemia.^60^

The REMOVAL study is a unique multi‐centre, double‐blind, randomised, placebo‐controlled trial that studied the cardiovascular and metabolic effects of metformin as an adjunct to insulin therapy in 493 patients with T1D over 3 years.^61^ The study failed to show an effect for metformin on the primary end point, atherosclerosis progression as measured by carotid intima‐media thickness (cIMT) and did not modulate glycaemia or insulin requirements. However, it reduced weight and demonstrated a modest yet significant reduction in LDL cholesterol. While this was a large and important study, it focused primarily on intermediate vascular outcomes, and it was not powered to analyse hard cardiovascular end points. A subsequent subgroup exploratory analysis of the REMOVAL study suggested that the effectiveness of metformin in slowing vascular pathology in T1D may be influenced by smoking status; metformin significantly reduced the progression of vascular disease in never‐smokers but had no significant effect in ever‐smokers.^62^

In a one‐year‐long placebo‐controlled trial involving 100 individuals with poorly controlled T1D (HbA1c ≥ 8.5%), metformin did not improve glucose control but did facilitate reductions in body weight and daily insulin requirements.^54^ The same study reported improvement in proatherogenic lipid profiles, independent of prior statin therapy.^63^

Another double‐blind randomised controlled trial (RCT) studied the effects of metformin on the cardiovascular system in 48 adolescents with T1D over 3 months (EMERALD trial) and showed that Metformin increased estimated glomerular filtration rate (eGFR), suggesting a potential renal benefit.^64^ A small pilot RCT study over 6 months on 42 uncomplicated T1D individuals showed that metformin improved vascular function irrespective of glycaemic control and body weight.^65^

A 10‐year retrospective study of three cohorts compared T1DM patients using adjuvant metformin for ≥6 months (*n* = 181), those who refused (*n* = 25) or used it for <6 months (*n* = 36), and a cross‐sectional cohort reference who were not offered this agent (*n* = 961). Initially, metformin users saw small, non‐significant decreases in body mass index (BMI) and insulin doses. However, after 10 years, there were no persistent effects on HbA1c, insulin dose or BMI. The study concluded that while metformin may have short‐term benefits in T1DM, no long‐term advantages are observed.^66^

Another retrospective study of 58 T1D individuals showed that metformin (allocated to half study participants) reduced insulin doses, independently of weight loss, at one year without a significant effect on HbA1c.^67^

A recent metanalysis evaluated the impact of metformin as an adjunct to insulin therapy in T1D^68^ and reported that this agent reduced both insulin requirements and weight and improved the lipid profile without affecting glycaemic control.

### 4.2 Potential risks of metformin

Gastrointestinal side effects, such as metallic taste, abdominal discomfort, nausea and diarrhoea, are the most common.^69^ In clinical trials (including those in T2D), approximately 5%–8% of study participants discontinue metformin because of gastrointestinal side effects.^70^ In a recent meta‐analysis of 404 T1D patients on metformin, there were 156 instances of gastrointestinal side effects (41%) with only 57 events of 400 individuals in the placebo group (14%) with an relative risk (RR) of 2.67 (95% confidence interval [CI] 2.06 to 3.45).^68^ There is also the potential risk of lactic acidosis, particularly in patients with renal impairment, but this remains very rare indeed.^71^ While theoretically the gastrointestinal side effects of metformin can be ameliorated by using a long‐acting preparation, strong evidence for a favourable side effect profile is lacking, although compliance certainly improves with the long‐acting preparation.^72^

Taken together, it appears that in the short term, metformin reduces insulin requirements, improves cardiometabolic markers without a clear effect on HbA1c. However, the limited data suggest that the beneficial effects of metformin are not sustained long term. While there is an increase in gastrointestinal side effects, there are no real safety concerns with this agent, and given the potential benefits, at least in the short term, metformin may be considered for the management of individuals with T1D who display features of IR.

Key studies on metformin use in T1D are summarised in Table 1.

**TABLE 1:** Key studies on metformin use in type 1 diabetes (T1D).

Study design and numbers	Duration	Primary Outcome(s)	Secondary outcome(s)	Reported side effects
RCT (REMOVAL)^61^ (*n* = 493 T1D)	3 years	Averaged mean carotid intima‐media thickness not significantly reduced	HbA1c: ↓ at 3 months but this was not sustained. Weight: ↓ by 1.2 kg Insulin Dose: no change LDL‐c: ↓ by 0.13 mmol/L eGFR: ↑ 4.0 mL/min per 1.73 m^2^	Gastrointestinal side effects leading to higher discontinuation in metformin than placebo group (27% and 12%, respectively) Hypoglycaemia was analysed and there was no increase in severe events with metformin
RCT^54^ (*n* = 100 T1DM; HbA1c ≥ 8.5%)	12 months, MF 1 gr bd	HbA1c: no difference at 12 months	Total daily insulin dose: ↓ by 5.7u/d Body Weight: ↓ by 1.74 kg with MF	Minor differences in gastrointestinal side effects. MF did not affect minor or major hypoglycaemia
RCT^63^ (*n* = 100 T1D)	12 months MF 1 gr bd	HbA1c: No change at 12 months	LDL‐c: ↓ by 0.3 mmol/L non‐HDL‐c: ↓ by 0.5 mmol/L	None reported
RCT^64^ (*n* = 48 youth T1D, aged 12–21 years)	3 months MF 1 gr bd	eGFR: ↑ by 13.9 mL/min/1.73 m2 versus placebo (remained significant after multivariable adjustments)	No differences in cystatin C, UACR or systemic inflammatory markers between the metformin and placebo groups	Numerical ↑ in gastrointestinal symptoms in the MF group Metallic taste with MF No increase in hypoglycaemia with MF
Pilot RCT^65^ (*n* = 42 T1D)	6 months, MF 850‐mg tds	Weight: ↓ by 2.27 FMD: Improved by 1.32% without a change in nitrate‐mediated Dilation	No changes in HbA1c, glycaemic variability or daily insulin dose	None of the patients enrolled had any side effects requiring a dose reduction. No episodes of severe hypoglycaemia
Retrospective study on T1D^66^ (on MF, ≥6 months, *n* = 181; refused or on MF <6 months, *n* = 62 and not offered MF, *n* = 961)	10‐year	BMI: Numerical ↓ with MF in early years but the effects were not sustained in the long‐term Insulin doses ↓ initially but this was not sustained HbA1c: No significant change with MF	‐	Not reported
Retrospective study on T1D^67^ (29 on MF/insulin 29 on insulin only)	12 months	MS: ↓ in number of individuals Insulin requirements: ↓ in the MF group Fasting & postprandial glucose: ↓ in the MF group	No differences in body weight, lipid profile or HbA1c comparing MF with the non‐MF group	Lactic acidosis and vitamin B12 deficiency were not observed during MF treatment ↑ gastrointestinal discomfort (17.2%) in MF users MF did not increase hypoglycaemia

Abbreviations: eGFR, estimated glomerular filtration rate; FMD, flow‐mediated arterial dilation; LDL‐c, low‐density lipoprotein cholesterol; MF, metformin; RCT, randomised controlled trial; UACR, urinary albumin creatinine ratio.

## 5 THIAZOLIDINEDIONES (TZDS)

Thiazolidinediones (TZDs), activators of the peroxisome proliferator‐activated receptor‐gamma (PPARγ), are recognised as insulin sensitisers.^73^

### 5.1 Clinical benefits of pioglitazone in T1D

Only a limited number of RCTs have explored the impact of pioglitazone in T1D with mixed outcomes. In one notable RCT, 60 lean adolescents with T1D had 30 mg of pioglitazone daily for six months. This treatment resulted in a significant reduction in HbA1c (−0.22 ± 0.29%) and improvement in postprandial plasma glucose levels. The proportion of participants achieving HbA1c ≤7% rose from 53% to 70% in the pioglitazone group, whereas no significant change was observed in the placebo group. Importantly, this study reported no differences in body weight, hypoglycaemic episodes, insulin requirements or lipid profiles.^74^

Another trial focused on 35 adolescents with suboptimal control of T1D and features of insulin resistance (insulin requirements >0.9 IU/kg/d), revealing no improvement in glycaemic control after six months of pioglitazone therapy. Disappointingly, there was an increase in BMI in the pioglitazone group compared with the placebo group.^75^

### 5.2 Potential risks of TZDs in T1D

Pioglitazone can cause fluid retention, thus increasing heart failure risk. It can also cause weight gain, partly due to fluid retention and partly to the expansion of adipose tissue. Some reports linked its use to a higher risk of fractures and reduced bone density, especially in postmenopausal women and those on glucocorticoids or proton pump inhibitors.^76^, ^77^

While pioglitazone appears to offer metabolic benefits, the side effect profile is not favourable and therefore the benefit:risk ratio is questionable, explaining why this agent is not usually considered for adjunctive therapy in T1D.

## 6 SODIUM‐GLUCOSE COTRANSPORTER‐2 INHIBITORS (SGLT2I)

SGLT2i, originally approved for T2D, has shown substantial promise in T1D through improving glucose profile while also promoting weight loss and reducing blood pressure.^78^

### 6.1 Clinical benefits of SGLT2i in T1D

The DEPICT‐1 and DEPICT‐2 trials, involving 833 and 813 T1D participants respectively, were randomised, double‐blind, multicentre studies which demonstrated that adjunctive treatment with dapagliflozin significantly reduced HbA1c compared to placebo at 24 weeks (0.20%–0.25% reduction with 5 mg/day and 0.25%–0.36% reduction with 10 mg/day). Also, a significant weight loss was documented ranging from 3.0% to 4.4% with 5 mg/d to 4.5%–4.9% with 10 mg/d.

The EASE (Empagliflozin as Adjunctive to inSulin thErapy) phase 3 programme, comprising two double‐blind, placebo‐controlled trials in T1D^79^ reported significant reductions in HbA1c at doses of 2.5 mg (−0.28%), 10 mg (−0.54%) and 25 mg (−0.53%), without an increase in hypoglycaemia. Furthermore, notable reductions were observed in patient weight, blood pressure and daily insulin requirements across the empagliflozin treatment arms.

Sotagliflozin in adults with T1D was evaluated in two phase 3 RCTs over one‐year period in two studies, inTandem1 and inTandem2.^80^, ^81^ The first, conducted in North America, randomised 793 participants to sotagliflozin 200 mg daily, 400 mg daily or placebo after an initial 6 weeks of insulin optimisation. Results showed significant reductions in HbA1c, weight and daily insulin doses with both doses of sotagliflozin, along with improvements in the Diabetes Treatment Satisfaction Questionnaire (DTSQ) scores. Similarly, the international inTandem2 trial, conducted mainly in Europe, involved 782 participants randomised to the same treatment regimens. Similarly to inTandem1, there was a significant reduction in HbA1c, weight, fasting glucose and insulin doses, with a 24‐week continuous glucose monitoring (CGM) sub‐study showing increased time in range and decreased postprandial excursions in glucose without an increase in hypoglycaemia.

This was followed by the InTandem 3 trial, which evaluated sotagliflozin in a global, phase 3, double‐blind study conducted at 133 centres.^82^ 1402 patients with T1D receiving insulin therapy were randomised to either sotagliflozin (400 mg per day) or placebo for 24 weeks. The primary outcome of achieving HbA1c <7.0% without severe hypoglycaemia or diabetic ketoacidosis was met by a significantly higher percentage in the sotagliflozin group (28.6%) compared to the placebo group (15.2%). Overall, HbA1c in the sotagliflozin group fell by 0.46%, associated with a decrease in weight (−2.98 kg), systolic blood pressure (−3.5 mm Hg) and average daily bolus insulin dose (−2.8 units) without an increase in hypoglycaemia; if anything, the rate of documented hypoglycaemia (3.1 mmol/L or lower) was significantly lower in the sotagliflozin group.

Other benefits of SGLT2 inhibitors, beyond their metabolic effects, are their potential impact on cardiovascular and renal health.^83^, ^84^, ^85^ However, studies have been conducted in T2D, and adequately powered hard clinical outcome trials in T1D are lacking. Given that T2D is characterised by IR, it is not unreasonable to assume that those with T1D and IR will have a similar benefit. Importantly, DEPICT trials, along with other studies (EASE and InTandem3 trials), have shown a reduction in albuminuria with the use of SGLT2i in T1D, suggesting that the renal protective effects of these agents are not only specific to T2D.^82^, ^86^, ^87^, ^88^

Importantly, the improvement in HbA1c with SGLT2i did not occur at the expense of increased hypoglycaemia,^89^, ^90^ representing a clear advantage over increased insulin doses for treating hyperglycaemia.

In a real‐world retrospective cohort study involving 992 individuals with T1D managed with SGLT2i and 1822 patients with T1D treated with GLP‐1 RA, both therapies resulted in significant reductions in HbA1c over a period of five years (reduction of 0.2% and 0.5%, respectively).^91^ The group receiving SGLT2i exhibited preservation of eGFR over the same timeframe (+3.5 mL/min per 1.73 m^2^), including among those with pre‐existing chronic kidney disease (CKD). Furthermore, the incidence of heart failure, CKD and hospitalisations for any reason was notably lower in the SGLT2i group compared to the GLP‐1 RA group, despite a higher baseline prevalence of heart failure, hypertension, IHD and CKD in the SGLT2i cohort. Another retrospective real‐world study across 2 European centres examined the impact of using SGLT2i in conjunction with insulin among 199 adults.^92^ Key findings over a 12‐month period included a decrease in average HbA1c by 0.5%, a reduction in weight by 2.9 kg, a decline in daily insulin dose by 8.5% and an improvement in renal function.

Other real‐world data from the German/Austrian DPV registry explored 12 months of adjunct SGLT2i treatment in 233 T1D patients^93^ and showed a significant reduction in HbA1c and blood pressure coupled with an improvement in lipid profile. Of note, none of the patients had a diabetic ketoacidosis (DKA) in the year before or during the 12 months of SGLT2i treatment.

It is worth noting that SGLT2i exhibit renoprotective properties by reducing intraglomerular pressure and attenuating hyperfiltration, primarily through the activation of tubuloglomerular feedback.^94^ Elevated intraglomerular pressure is a key factor in renal hyperfiltration, which is evident to be as high as 60% in individuals with T1D, contributing to the onset and progression of diabetic renal disease.^95^ It has been reported that short‐term administration of SGLT2i (i.e. empagliflozin for 8 weeks) markedly decreases hyperfiltration, thereby mitigating hyperfiltration‐induced renal damage.^96^

In summary, SGLT2i are effective in T1D by improving glycaemic control, facilitating weight loss and lowering blood pressure. Although primarily tested in T2D, emerging data suggest potential cardiovascular and renal benefits in T1D. They also reduce the hypoglycaemia risk, enhancing their suitability as an adjunct therapy.

It is worth noting that SGLT2i treatment also showed improvement in quality‐of‐life measures.^97^, ^98^ Therefore, SGLT2i treatment in T1D has metabolic, potential vascular and patient‐focused benefits.

### 6.2 Potential risks of SGLT2i in T1D

Three meta‐analyses have shown that SGLT2i increase the rate of diabetic ketoacidosis (OR 3.38) and genital tract infection (OR 3.44) in those with T1D.^99^, ^100^, ^101^ In general, studies have reported a higher incidence of DKA in T1D patients treated with SGLT2i compared to those on placebo.^102^

Suggested mechanisms include inhibition of glucose reabsorption in proximal renal tubules by SGLT2i, prompting a metabolic shift from glucose to lipid utilisation, which can increase ketone production in the liver. This increase is partly due to elevated glucagon levels from direct effects of SGLT2i on pancreatic α‐cells and reduced renal clearance of ketone bodies, with the net effect of increased plasma ketones.^103^ SGLT2i can also result in volume depletion and dehydration, which further exacerbate ketoacidosis. Precipitating factors leading to SGLT2i‐associated ketoacidosis include reduction in insulin doses (or omission), alcohol excess and low‐carbohydrate diets. To mitigate these risks, careful patient selection, education and monitoring are essential. Ketone risk mitigation protocols exist, and one such protocol is the Strategic Training Initiative for the Prevention of Hyperglycaemia (STICH), which recommends regular monitoring of ketone levels, especially during illness or when glucose levels are consistently high.^104^ It also recommends immediate interventions upon high ketone detection, including temporary cessation of SGLT2i, insulin administration, increased carbohydrate intake and adequate hydration.

Urinary tract infections and mycotic infections are less of a concern but should still be closely monitored and patients educated appropriately.^105^ Fournier's gangrene is a serious complication of these therapies, but fortunately, it is very rare, particularly in those with type 1 diabetes.^106^

Key studies on SGLT2i use in T1D are summarised in Table 2.

**TABLE 2:** Key studies on Sodium‐Glucose Co‐Transporter 2 inhibitors (SGLT2i) use in type 1 diabetes (T1D).

Study design and numbers	Duration	Primary outcome(s)	Secondary outcome(s)	Reported side effects
RCT (DEPICT‐1).^86^ (*n* = 833 T1D; majority of European descent; HbA1c 7.5%–10.5%)	52 weeks (24‐week short‐term and 28‐week extension period). Dapagliflozin 5 mg, or 10 mg, or placebo.	Dapagliflozin 5 mg: ↓ HbA1c by −0.33% ↓ body weight by −2.95%. Dapagliflozin 10 mg: ↓ HbA1c by −0.36% ↓ body weight by −4.54%.	More patients in the dapagliflozin groups achieved an HbA_1c_ reduction ≥0.5%, without an episode of severe hypoglycaemia. ↓ fasting plasma glucose in the dapagliflozin 5‐mg and 10‐mg groups compared with placebo. ↓ SBP across all groups at week 52 (HTN background), dapagliflozin groups had a greater reduction than the placebo group	Comparable hypoglycaemia events across all groups, ↑ DKA in dapagliflozin groups (4.0% for 5 mg, 3.4% for 10 mg, versus 1.9% in placebo).
RCT DEPICT‐2.^87^ (*n* = 813 T1D; Asia‐Pacific region: mainly Japan).	52 weeks (24‐week short‐term and 28‐week extension period). (dapagliflozin 5, 10 mg, or placebo)	Dapagliflozin 5 mg: ↓ HbA1c by −0.20% ↓ body weight reduction of −4.42%. Dapagliflozin 10 mg: ↓ HbA1c by −0.25% ↓ body weight by −4.86%.	More patients in the dapagliflozin groups achieved an HbA_1c_ reduction ≥0.5% (5.5 mmol/mol), without an episode of severe hypoglycaemia. ↓ SBP in the dapagliflozin treatment groups compared with the placebo group at week 52.	Serious adverse events occurred in 11.8% of patients in the dapagliflozin 5‐mg group, 7.0% in the 10‐mg group, and 5.9% in the placebo group. Hypoglycaemic events were similar across all groups. Definite DKA events were more frequent in dapagliflozin groups compared to placebo (4.1% in 5 mg, 3.7% in 10 mg, vs. 0.4% in placebo).
RCT (EASE trial),^79^ (*n* = 1707 T1D) (EASE2 & EASE3)	EASE‐2 for 52 weeks; empagliflozin 10 mg, 25 mg or placebo EASE‐3 for 26 weeks, empagliflozin 2.5 mg, 10 mg, 25 mg or placebo.	↓ HbA1c: −0.28% (with 2.5 mg), −0.54% (10 mg), and − 0.53% (25 mg). ↓ Weight: −1.8 kg (2.5 mg), −3.0 kg (10 mg), −3.4 kg (25 mg). ↑ TIR: +1.0 h/day (2.5 mg), +2.9 h/day (10 mg), +3.1 h/day (25 mg).	↓ total daily insulin dose: −6.4% (2.5 mg), −13.3% (10 mg), −12.7% (25 mg). ↓ SBP ‐2.1 mmHg (2.5 mg), −3.9 mmHg (10 mg), −3.7 mmHg (25 mg).	Genital infections occurred more frequently with empagliflozin. DKA rate 4.3% (10 mg), 3.3% (25 mg), 0.8% (2.5 mg), and 1.2% (placebo). Severe hypoglycaemia was rare and similar between empagliflozin and placebo groups.
RCT (inTandem1),^80^ (*n* = 793 T1D; North America)	24 weeks for primary end points, extended to 52 weeks. Sotagliflozin 200 mg, 400 mg, placebo).	Significant ↓ HbA1c: At 24 weeks: ○ ↓ 0.36% with 200 mg ○ ↓ 0.41% with 400 mg At 52 weeks: ○ ↓ 0.25% with 200 mg ○ ↓ 0.31% with 400 mg	At 52 weeks (sotagliflozin 400 vs placebo): ↓ Fasting plasma glucose by 1.08 mmol/L ↓ Weight by 4.32 kg ↓ Bolus insulin dose by 15.63% ↓ Basal insulin dose by 11.87%. At 24 weeks: ↑ Diabetes Treatment Satisfaction Questionnaire scores by 2.5 points.	Genital mycotic infections and diarrhoea more frequent with sotagliflozin. DKA rates: 3.4% in patients on sotagliflozin 200 mg. 4.2% in patients on sotagliflozin 400 mg. 0.4% in patients on placebo. Severe hypoglycaemia: 6.5% in each sotagliflozin group (200 mg & 400 mg). 9.7% in placebo.
RCT (inTandem 2),^81^ (*n* = 782 T1D)	24 weeks (primary end point), also extended to 52 weeks. (Sotagliflozin 200 mg, 400 mg, placebo).	Significant ↓ HbA1c: At 24 weeks: 0.37% with 200 mg 0.35% with 400 mg Differences maintained at 52 weeks	At 52 weeks, proportion of patients with HbA1c < 7.0%, no severe hypoglycaemia, and no DKA: Sotagliflozin 200 mg: 25.67% Sotagliflozin 400 mg: 26.62% Placebo: 14.34% (*p* ≤ 0.001) At 52 weeks: Sotagliflozin 400 mg: ↓ Fasting plasma glucose: −0.87 mmol/L ↓ Weight: −2.92 kg ↓ Total daily insulin dose: −8.2% ↑ Treatment satisfaction increased, ↓ diabetes distress with sotagliflozin	At 52 Weeks: ↓ documented hypoglycaemia with sotagliflozin. Severe hypoglycaemia: ○ Placebo: 5.0% ○ Sotagliflozin 200 mg: 5.0% ○ Sotagliflozin 400 mg: 2.3% DKA Placebo: 0% Sotagliflozin 200 mg: 2.3% Sotagliflozin 400 mg: 3.4%
RCT, (InTandem3),^82^ (*n* = 1402 T1D).	24 weeks Sotagliflozin	Achieving HbA1c < 7.0% without severe hypoglycaemia or DKA: Sotagliflozin: 28.6% Placebo: 15.2%	Sotagliflozin: ↓ HbA1C by 0.46 percentage points, ↓ weight by 2.98 kg, ↓ SBP by 3.5 mmHg ↓ Mean Daily Bolus Insulin Dose by 2.8 units/day.	DKA ↑ in sotagliflozin: 3.0% versus placebo: 0.6%. Severe Hypoglycaemia: Similar rates: Sotagliflozin 3.0% versus Placebo 2.4%. ↓ Hypoglycaemia in the sotagliflozin group.
Retrospective cohort study,^91^ (*n* = 992 SGLT2i, *n* = 1822 GLP‐1RA)	5 years after initiation of therapy.	↓ HbA1c: SGLT2i: ↓−0.2%) GLP‐1 RA: ↓−0.5% eGFR Over 5 Years: SGLT2i: ↑ +3.5 mL/min per 1.73 m^2^ GLP‐1 RA: ↓−7.2 mL/min per 1.73 m^2^	Heart Failure: ○ ↓risk in SGLT2i (RR 0.44 [95% CI 0.23, 0.83], *p* = 0.0092) CKD: ○ ↓risk in SGLT2i (RR 0.49 [95% CI 0.28, 0.86], *p* = 0.0118) Hospitalisation: ○ Less likely in SGLT2i (RR 0.59 [95% CI 0.46, 0.76], *p* ≤ 0.0001)	DKA ○ ↑ in SGLT2i (RR 2.08 [95% CI 1.05, 4.12], *p* = 0.0309) Urinary Tract Infection/Pyelonephritis: ○ ↑ in SGLT2i cohort (RR 2.27 [95% CI 1.12, 4.55], *p* = 0.019)
Retrospective cohort study^92^;. (*n* = 199 T1D)	12 months	HbA1c: ↓−0.5% Weight: ↓−2.9 kg Daily insulin: ↓−8.5% Greatest HbA1c reduction in patients with baseline HbA1c >8%: −0.7% (64 mmol/mol) Most significant weight loss in subjects with BMI >27 kg/m^2^: −3.5 kg	eGFR ↑in individuals with baseline <90 mL/min/1.73 m^2^: +4.5 mL/min/1.73 m^2^ UACR ↓ in those with >15 mg/g: −16.6 mg/g	Genital infections: 22.6% Ketosis episodes: 2.5% DKA 3.5% No severe hypoglycaemia events.
Real‐world data,^93^ (*n* = 233 T1D)	Data collected over 8 years, from 2012 to 2020.	12‐Month Outcomes Post SGLT2i Initiation: Significant reductions in ↓ HbA1c by 0.63% ↓ DBP by 1.87 mmHg ↓ Cholesterol by 8.71 mg/dL ↓ LDL Cholesterol by 5.58 mg/dL Small ↓ SBP by 2.15 mmHg	No Significant Changes Observed in mean BMI, Mean insulin dose/kg, HDL‐Cholesterol, Triglycerides, eGFR, proportion of individuals with microalbuminuria	Similar severe Hypoglycaemia incidence prior to SGLT2i initiation and during the first 12 months on SGLT2i at 3% DKA: None either in the year before or during the 12 months of SGLT2i treatment (0%).

Abbreviations: CKD, chronic kidney disease; DBP, diastolic blood pressure; DKA, diabetic ketoacidosis; eGFR, estimated glomerular filtration rate; GLP1RA, Glucagon‐Like Peptide‐1 receptor agonists; HTN, hypertension; RCT, randomised controlled trial; SBP, systolic blood pressure; SGLT2i, Sodium‐Glucose Co‐Transporter 2 inhibitors; TIR, time in range; UACR, urinary albumin creatinine ratio.

## 7 GLUCAGON‐LIKE PEPTIDE‐1 RECEPTOR AGONISTS (GLP‐1RA)

GLP‐1 receptor agonists suppress glucagon release, slow gastric emptying and promote satiety, explaining their favourable effects on glycaemia and weight in T1D.^107^ GLP‐1 also enhances insulin secretion through the activation of the cAMP response element‐binding protein (CREB), pivotal for the transcription of the insulin gene, while also activating protein kinase A (PKA), which is responsible for phosphorylating target proteins that promote insulin secretion. Moreover, the phosphoinositide 3‐kinase (PI3K)/Akt signalling pathway is also activated by GLP‐1, crucial for the survival and proliferation of pancreatic β‐cells. The activation of Akt not only stimulates β‐cell proliferation and reduces apoptosis but also enhances insulin secretion by regulating key downstream effector molecules, including FoxO and the glucose transporter type 2 (GLUT2).^108^ In addition to glucose levels, GLP‐1RAs modulate AMP‐activated protein kinase (AMPK) pathways, enhancing fatty acid oxidation—a crucial step for improving lipid profile and reducing ectopic fat deposition. This is achieved by inhibiting acetyl‐CoA carboxylase, which in turn decreases malonyl‐CoA levels, restraining the inhibition of carnitine palmitoyltransferase 1 (CPT1), the primary enzyme involved in the mitochondrial uptake of fatty acids.^109^

### 7.1 Clinical benefits of GLP‐1RA

These agents increase food‐mediated insulin secretion while simultaneously inhibiting inappropriate glucagon secretion. The former mode of action is irrelevant in T1D, but the effects on glucagon may have a role in improving glucose levels. ADJUNCT‐ONE and ADJUNCT‐TWO were key randomised controlled phase 3 trials in 1398 and 835 participants with T1D treated with liraglutide (0.6, 1.2 or 1.8 mg) or placebo, all as adjuncts to insulin. Liraglutide significantly reduced HbA1c by 0.3%–0.5% compared to placebo,^110^, ^111^ while significantly reducing mean body weight by −2.2 to −5.1 kg, effects which were dose‐dependent. Significant reductions in daily insulin dose and increased quality of life were also observed in the liraglutide groups compared with placebo.

In a preliminary small study of 10 newly diagnosed T1D individuals, semaglutide eliminated the need for mealtime insulin doses among the entire study cohort and allowed 70% to stop basal insulin within 6 months, with the mean HbA1c decreasing from 11.7% at baseline to 5.9% at 6 months and 5.7% at 12 months.^112^ While these results are promising, larger studies are required, including an appropriate control group with a longer follow‐up period to further characterise possible temporary remission of T1D with this agent.

In a retrospective chart review conducted in the United States and Western Europe, the effectiveness of semaglutide was assessed in 50 overweight or obese patients with T1D.^113^ These patients were matched with a control group of 50 individuals not on any weight loss medications. The study, which tracked outcomes over a year, reported significant improvements in the semaglutide group, including a reduction in BMI by an average of 7.9%, and a better glycaemic profile as indicated by lowerHbA1c, reduced glucose variability and increased time in glucose range (TIR).

A retrospective study included 11 patients with T1D with detectable C‐peptide and positive glutamic acid decarboxylase (GAD) who were treated consecutively with GLP‐1RAs alongside insulin (nine patients had liraglutide, two patients had dulaglutide).^114^ After 12 weeks of therapy, HbA1c decreased from 10.74 ± 0.96% to 7.4 ± 0.58% (*p* < 0.01), total insulin dose was reduced by 64% from 33 ± 6 units to 11 ± 5 units (*p* < 0.01) and C‐peptide concentrations rose from 0.43 ± 0.09 ng/mL to 1.42 ± 0.42 ng/mL (*p* = 0.01). Additionally, body weight showed a trend towards a decrease from 71 ± 2.0 kg to 69 ± 2 kg (*p* = 0.06). Remarkably, half of the patients no longer required insulin therapy by the end of the study period.

It should be noted that studies on adjunctive therapies have generally focused on glycaemia when other metabolic markers could be equally important, particularly in relation to the amelioration of IR secondary to weight loss.^115^, ^116^

Of note, a number of meta‐analyses advocate the use of GLP‐1RA as adjunctive therapy for patients with T1D for weight loss, insulin dose reduction, glycaemic control and enhancing metabolic profile without an increased risk of serious adverse events.^117^, ^118^, ^119^ Also, the reduction in exogenous insulin requirements by GLP‐1RA may help to mitigate the risk of severe hypoglycaemic events, thereby improving overall treatment safety and patient quality of life.

It is worth noting that there are several registered and ongoing placebo‐controlled trials evaluating the efficacy and safety of semaglutide in T1D. Examples include the RESET1 Trial (ACTRN12623001277639) – recruiting 60 individuals with T1D and assessing the effects of semaglutide on arterial stiffness as the primary endpoint. The ADJUST T1D Trial (NCT05537233) is also investigating semaglutide in T1D but focused on those with a BMI > 30 kg/m^2^ and having detailed glycaemic endpoints. The Semaglutide Trial (NCT06411210) has largely similar inclusion criteria to ADJUST T1D but explores a higher dose of semaglutide (2.4 mg once/week) and investigates the effects on glycaemia and weight. In addition to glycaemic and vascular health studies, the RT1D Trial (NCT05822609) is investigating the effects of semaglutide on renal disease progression in individuals with T1D.

Tirzepatide, a novel dual receptor agonist for glucagon‐like peptide‐1 (GLP‐1) and glucose‐dependent insulinotropic polypeptide (GIP), has shown considerable promise in managing T2D by reducing HbA1c and facilitating weight loss through a number of mechanisms.^120^, ^121^

GIP is a potent stimulator of glucose‐dependent insulin secretion, and it regulates metabolic processes in adipose tissue, such as lipolysis and lipogenesis. By increasing metabolic flexibility, GIP allows for greater fat utilisation in the fasting state and decreases fat availability postprandially. When combined with GLP1‐RA, GIP agonism provides synergistic effects that significantly improve the regulation of energy balance. This synergy extends to cell‐surface receptor signalling within the brain and adipose tissue, further enhancing drug efficacy.^122^ This synergy between GLP‐1RA/GIP explains the potential superior metabolic efficacy of tirzepatide compared with standalone GLP1‐RA therapies.^123^, ^124^

Interestingly, the SURPASS‐1 trial, tirzepatide as monotherapy for early T2D, demonstrated significant improvements in insulin sensitivity and multiple biomarkers of pancreatic beta‐cell function, and potentially preservation.^122^ This can be linked to the action of GLP‐1 and GIP on their unique receptors in pancreatic *β*‐cells, promoting *β*‐cell proliferation with inhibition of cellular apoptosis and expansion of *β*‐cell mass.^125^

No RCTs have studied the effects of this tirzepatide in T1D, although a few registered trials are ongoing. For example, a phase 2 trial is ongoing on 40 patients with T1D comparing insulin alone with the combination of insulin and tirzepatide (NCT06180616). The primary aim of the trial is to determine whether adjunctive treatment with tirzepatide over 32 weeks reduces body weight in patients with T1D who are overweight or obese, compared to those receiving insulin‐only therapy. Secondary objectives include improvement in glycaemia (HbA1c, time in range) decrease in insulin requirements and reduction in comorbidities associated with obesity and T1D. The TIRTLE trial (ACTRN12624000111572) is another ongoing trial, which is exploring the impact of tirzepatide on glycaemic measures and weight in T1D. The outcomes of theses RCTs could reshape treatment protocols by supporting the use of dual GLP‐1/GIP agonists in the management of T1D complicated by obesity.

A retrospective observational study on 26 obese and overweight adults with T1D demonstrated a significant drop in HbA1c by 0.45% and 0.59% at 3 and 8 months respectively after initiating tirzepatide,^126^ coupled with a 10.1% weight loss at 8 months. The decrease in HbA1c was associated with improved CGM‐derived glucose metrics, including increased TIR and time in tight target range, along with a decrease in time above range without a significant increase in hypoglycaemia, indicating improved glycaemic stability. Another retrospective study, presented at the American Diabetes Association and yet to be fully published, involved 52 adults with T1D treated with tirzepatide primarily for obesity^127^ and showed significant weight loss with reductions of 6%, 8% and 14% in total body weight at 3, 6 and 12 months, respectively. At 6 months, a 1% reduction in HbA1c was documented together with a 29% increase in TIR and a 32% decrease in total daily insulin dose. While no severe episodes of hypoglycaemia or DKA were reported, side effects occurred in a quarter of patients, with nausea being the most common (15%). An additional retrospective off‐label study^128^ explored tirzepatide in 62 adult obese and overweight T1DM patients. It concluded that tirzepatide facilitated an average 18.5% weight loss and improved glucose control at 1 year.

### 7.2 Potential risks of GLP‐1RA in T1D

While gastrointestinal side effects with these agents, such as nausea and vomiting, are common, they are generally mild and transient. There is a minor concern regarding the potential risk of pancreatitis and thyroid C‐cell tumours, although these risks appear to be low based on current evidence, and recent studies have not reported such complications.^105^, ^129^, ^130^

The impact of GLP‐1RA on diabetic retinopathy remains a topic of debate. While GLP‐1 receptors exist in the human retina and some studies suggest GLP‐1RA may be neuroprotective,^131^ concerns linger that it may exacerbate existing diabetic retinopathy, as shown in the SUSTAIN‐6 trial.^132^ However, earlier SUSTAIN 1 to 5 trials with semaglutide showed no such effect.^133^, ^134^, ^135^ A recent study of 692 GLP‐1RA users failed to find an association between GLP‐1RA and retinopathy.^136^ Of note, all studies involved T2D patients, and current evidence does not suggest worsening retinopathy with GLP‐1RA in T1D.

Sharing the above potential risks with GLP1‐RA, the long‐term safety profile of tirzepatide in T1D remains unclear. A recent systematic review and metanalysis on the safety of tirzepatide showed that severe hypoglycaemia, fatal adverse events, acute pancreatitis, cholelithiasis and cholecystitis are rare.^137^ The mild increase in hypoglycaemic risk can be related to insulin co‐administration and therefore insulin doses should be closely monitored in individuals with T1D having these adjunctive therapies.

Key studies on GLP‐1RA use in T1D are summarised in Table 3.

**TABLE 3:** Key studies on Glucagon‐Like Peptide‐1 receptor agonists (GLP1‐RA) use in type 1 diabetes (T1D).

Study design and numbers	Duration	Primary outcome(s)	Secondary outcome(s)	Reported side effects
RCT (ADJUNCT‐One)^110^ (*n* = 1398 T1D)	52 weeks, Liraglutide (1.8, 1.2 or 0.6 mg)	HbA1c: ↓ by 0.34–0.54% Significant ↓ for liraglutide 1.8 mg by −0.20%, and 1.2 mg by −0.15% compared to placebo. Insulin dose: ↓ insulin dose with liraglutide 1.8 mg by 0.92, and liraglutide 1.2 mg by 0.95 compared to placebo.	Body weight: Mean body weight significantly reduced in all liraglutide groups: 1.8 mg: ↓ −4.9 kg 1.2 mg: ↓ −3.6 kg 0.6 mg: ↓ −2.2 kg	Hypoglycaemia: ↑ rates of symptomatic hypoglycaemia in all liraglutide groups: 1.8 mg: ↑ 1.31 [95% CI 1.07; 1.59]. 1.2 mg: ↑ 1.27 [95% CI 1.03; 1.55]. 0.6 mg: ↑ 1.17 [95% CI 0.97; 1.43]. Hyperglycaemia with Ketosis: Significantly ↑ for liraglutide 1.8 mg by 2.22% Pancreatitis: only one case in the liraglutide 0.6 mg group
RCT (ADJUNCT‐2)^111^ (*n* = 835 T1D)	26‐week Liraglutide (1.8, 1.2 or 0.6 mg)	HbA1C Significant ↓ with liraglutide compared to placebo: 1.8 mg: ↓ −0.33% 1.2 mg: ↓ −0.22% 0.6 mg: ↓ −0.23% Placebo: ↑ +0.01%	Body Weight: Significant reduction in mean body weight: 1.8 mg: ↓ −5.1 kg 1.2 mg: ↓ −4.0 kg 0.6 mg: ↓ −2.5 kg Placebo: ↓ −0.2 kg Insulin Dose: Significant ↓ in daily insulin dose Quality of Life: Significant ↑ in quality of life were observed with liraglutide compared to placebo.	Symptomatic Hypoglycaemia: ↑ rates for liraglutide 1.2 mg versus placebo: 21.3 versus 16.6 events/patient/year (*p* = 0.03) Hyperglycaemia with Ketosis: ↑ for liraglutide 1.8 mg versus placebo: 0.5 versus 0.1 events/patient/year (*p* = 0.01)
Preliminary observational retrospective analysis^112^ (*n* = 10 T1D)	1 year Semaglutide	Insulin Requirements: Prandial insulin eliminated in all patients within 3 months. Basal insulin eliminated in 7 patients within 6 months. Doses maintained throughout the 12‐month follow‐up period.	Glycaemic Control: Mean HbA1c levels: ○ 5.9 ± 0.3% at 6 months ○ 5.7 ± 0.4% at 12 months Fasting C‐peptide levels ↑ to a mean of 1.05 ± 0.40 ng/mL. TIR achieved was 89 ± 3%.	Hypoglycaemia and Safety: Mild hypoglycaemia recorded during period of semaglutide dose increase. No episodes of hypoglycaemia post‐dose stabilisation. No episodes of DKA or other serious side effects reported.
Retrospective chart pilot study^113^ (*n* = 50 overweight or obese T1D)	1 year Semaglutide	BMI: ↓ of 7.9% ± 2.6% Body weight: ↓ of 15.9 lbs. ± 5.4 lbs. HbA1c: Significant ↓ TIR: Significant ↑	No changes in insulin dose, TAR, TBR compared to the control group.	None reported severe hypoglycaemia or DKA that needed hospitalisation
Retrospective analysis^114^ (*n* = 11 normal weight patients	12 ± 1 weeks (*n* = 9 liraglutide, n = 2 dulaglutide)	Glycaemic Control Improvements: HbA1c ↓ from 10.74 ± 0.96% to 7.4 ± 0.58% (*p* < 0.01). Body Weight Changes: ↓ from 71 ± 2.0 kg to 69 ± 2 kg (*p* = 0.06). Insulin Dose Reduction: ↓ by 64% from 33 ± 6 units to 11 ± 5 units (*p* < 0.01). Five out of 10 patients did not require insulin.	C‐Peptide Concentrations: ↑ significantly by 3.5‐fold from 0.43 ± 0.09 ng/mL to 1.42 ± 0.42 ng/mL (*p* = 0.01). Patients reported subjective improvements in quality of life	No increase in the incidence of subjective hypoglycaemia. Transient nausea reported in both groups. One patient from liraglutide discontinued treatment due to nausea after the first week.
Retrospective observational study^126^ (*n* = 26 obese and overweight adults)	8 months Tirzepatide	Significant HbA1c Reductions: ↓ 0.45% at 3 months ↓ 0.59% at 8 months Significant Body Weight Reductions: ↓ 3.4% at 3 months ↓ 10.5% at 6 months ↓ 10.1% at 8 months	Glucose Control Improvements: TIR 70–180 mg/dL: ↑ by 12.6% (*p* = 0.002) TITR 70–140 mg/dL: ↑ by 10.7% (*p* = 0.0016) TAR >180 mg/dL): ↓ by 12.6% (*p* = 0.002)	Relatively safe and well tolerated. Only 2 patients discontinued medication due to adverse effects.
Retrospective study^127^ (*n* = 52 T1D)	12 months Tirzepatide	Significant ↓ Total Body Weight Loss (TBWL): ↓ 6% [3–9] at 3 months (*n* = 44) ↓ 8% [5–15] at 6 months (*n* = 29) ↓ 14% [7–22] at 12 months (n = 13)	Significant changes from Baseline to Last Follow‐Up: TBWL%: ↓ of 8% HbA1c: ↓ of 1% TDD of insulin: ↓of 32% TAR ↓ of 28% [ TIR ↑ 29% [3–55] TBR ↓ of 32% showing a trend towards significance (*p* = 0.08)	No episodes of severe hypoglycaemia or DKA Incidence of side effects was 26%, the most common was nausea (15%). Two patients (4%) discontinued tirzepatide due to side effects.
retrospective single‐centre real‐world study^128^ (*n* = 62 overweight and obese T1D)	1‐year Tirzepatide	BMI and Weight Reductions: Significant ↓ BMI and ↓ weight in the Tirzepatide group compared to controls at all time points. Average weight loss of 18.5% (>46 pounds) HbA1c Reductions: ↓ observed as early as 3 months, sustained through 1 year (−0.67% at 1 year).	Insulin Dose Adjustments: ↓ starting at 3 months and continued throughout the study period. Glucose Monitoring Metrics: Significant ↑in mean glucose, TIR, TAR, standard deviation and coefficient of variation (CV).	No reported hospitalisations due to severe hypoglycaemia or DKA.

Abbreviations: DKA, diabetic ketoacidosis; RCT, randomised controlled trial; TAR, time above range; TIR, time in range; TITR, time in tight range.

## 8 A PRAGMATIC CLINICAL APPROACH FOR ADJUNCTIVE THERAPIES IN T1D

It is currently unknown who should undergo adjunctive therapy in the T1D cohort, and the first step should be the identification of patients who are likely to benefit. Those with high insulin requirements or elevated BMI are potential targets, but treatment cut‐offs are open to interpretation. Given the documented relationship between glucose disposal rate (eGDR) and adverse clinical outcomes, and the ease by which this IR marker can be calculated, it is not unreasonable to suggest using eGDR for deciding on adjunctive therapy. The cut‐off can be debated but given current data,^25^ T1D individuals with eGDR <6 mg/kg/min are perhaps the group to consider, provided they are concordant with their insulin therapies.

While current evidence suggests that metformin fails to offer beneficial vascular effects in the long term, its relatively favourable safety profile makes it a valuable adjunctive therapy for T1D patients who are insulin resistant, to enhance insulin sensitivity, reduce weight and insulin requirements, at least in the short term.

Effective patient selection is crucial to maximise the benefits of adjunctive therapies in T1D. Patients best suited for such therapies often exhibit specific characteristics beyond just inadequate glucose levels. These include, but are not limited to, a history of cardiovascular events, a significant degree of insulin resistance and/or persistent challenges with weight management. Identifying these patients requires a detailed assessment followed by a balanced clinical decision, which is not necessarily that simple given the limited evidence to date. Therefore, adjunctive therapies in T1D are largely “personalised” with initiation based on evidence from small studies while stop/continue decisions are based on response to these therapies. Another area that remains unclear is when to start adjunctive therapies in T1D. In those with established complications, particularly in the presence of insulin resistance, starting adjunctive therapies is a logical step to prevent further deterioration. However, it can be argued that adjunctive therapies should be started earlier to prevent complications from occurring in the first place. The counter‐argument is that the long‐term safety of adjunctive therapies in T1D is still unknown and therefore any decision around these treatments should be taken with great care and after full discussion with the patient.

In clinical practice, introducing metformin should start with a low dose to minimise gastrointestinal side effects, gradually increasing to the optimal dose. Given the short‐term benefits of metformin, health care professionals should closely monitor the response to this treatment, with a replacement agent introduced at an appropriate time.

SGLT2i have a favourable glycaemic profile and induce weight loss, while also potentially having renal and cardiac protective effects, although definitive outcome studies are lacking in T1D. They can be a valuable addition in T1D, provided there are no concerns over concordance with insulin therapy and there is a full understanding of DKA risk and ways of mitigating risk by the patient. Other risks include urinary and genital infections, which may preclude use in some patients. Fournier's gangrene is very rarely reported in T1D.

GLP‐1 RAs offer potential cardiovascular benefits in T1D given the favourable metabolic effects. Health care professionals should consider GLP‐1 RAs for T1D patients struggling with weight management while educating patients on gastrointestinal side effects and using this agent with caution in those with a history of pancreatitis. In individuals with rapid improvement in glycaemia following the start of these agents, early retinal screening is advocated, given findings in those with T2D, at least until the relationship between retinal changes and treatment with these agents is better understood.

**FIGURE 3:**
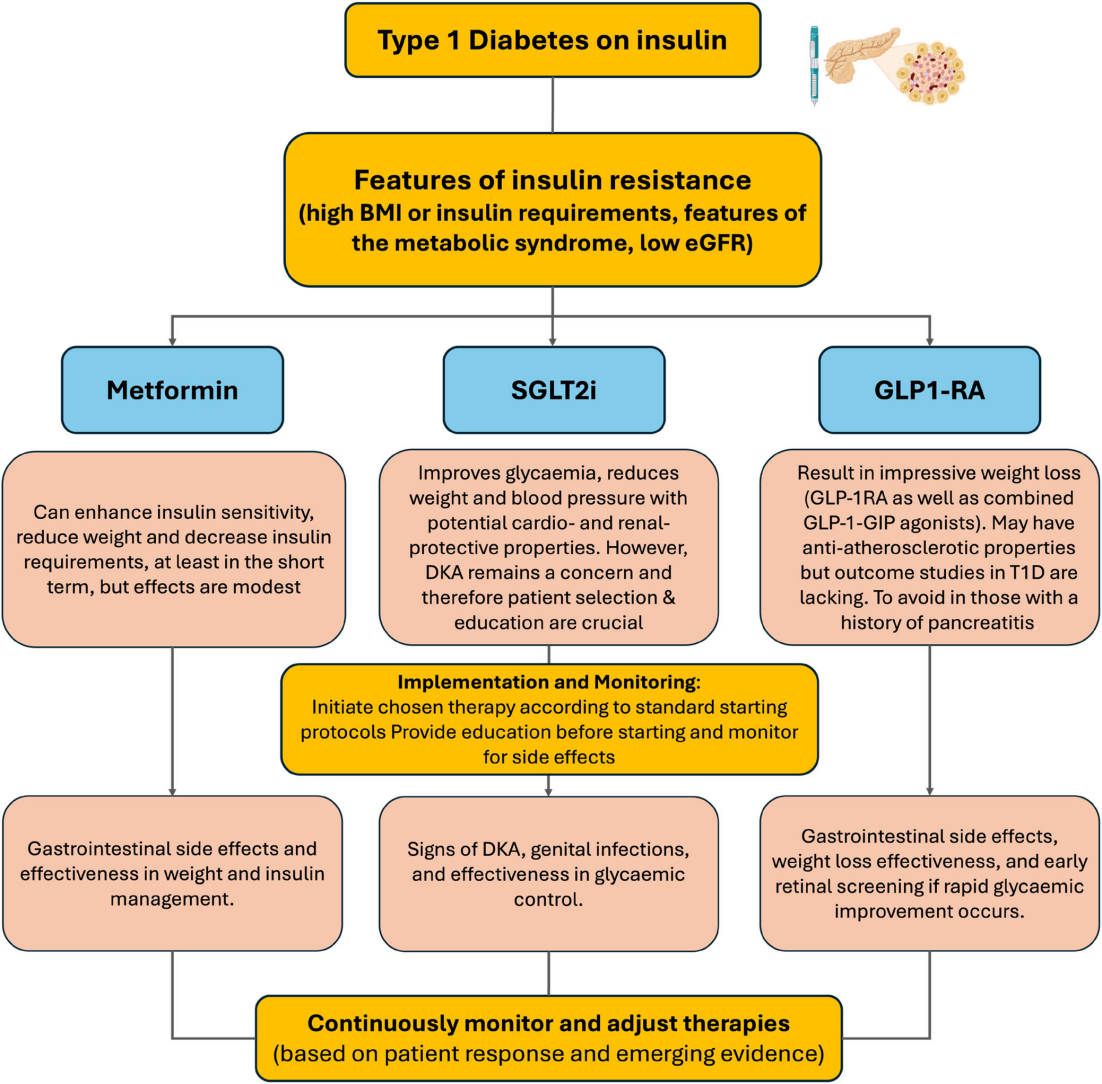
Strategic Overview of Adjunctive Therapies in the Management of Type 1 Diabetes with Suboptimal Control. This diagram presents a pragmatic approach for integrating Metformin, Sodium‐Glucose Co‐Transporter 2 inhibitors (SGLT2i) and Glucagon‐Like Peptide‐1/Glucose‐Dependent Insulinotropic Polypeptide receptor agonists (GLP‐1/GIP RAs) receptor agonists into the treatment of individuals with type 1 diabetes (T1D). The assessment includes insulin dosing, the presence of insulin resistance and concerns over cardiovascular and renal health. Metformin is recommended for enhancing insulin sensitivity and managing weight, whereas SGLT2i are noted for their potential to improve both glycaemic control and renal/cardiovascular outcomes. GLP‐1/GIP receptor agonists are highlighted for their role in weight management and potential cardiovascular benefits. The implementation and monitoring section outlines the initiation process, ongoing monitoring and adjustment of therapies based on individual patient responses and emerging clinical evidence. Each therapy is accompanied by a brief note on specific considerations or side effects to guide clinical decisions.

Incorporating these adjunctive therapies into T1D management requires a personalised approach, considering the patient's overall health, associated comorbidities, general understanding and lifestyle (Figure 3). It should be noted that the use of glycaemic adjunctive therapies in T1D is outside the licensed indications of these agents, which should be explained to patients before starting such treatment.

The combined use of different adjunctive therapies presents an intriguing opportunity for maximising benefits and improving short‐ and long‐term outcomes. However, given the lack of meaningful studies investigating combined adjunctive therapies, this remains an area where clinical judgement rather than solid evidence dictates the approach. Clinical trials designed to investigate combined adjunctive therapies, either from the start or using a step‐wise escalation, are needed to understand efficacy and safety. Moreover, identifying appropriate biomarkers, beyond weight and glycaemia, should help guide therapy escalation/combination, and this remains an area for future research.

## 9 GAPS IN KNOWLEDGE: SCOPE OF FUTURE RESEARCH

Substantial gaps in knowledge persist on the long‐term efficacy and safety of adjunctive therapies in T1D.

The lack of outcome data with adjunctive therapies is a major knowledge gap. Adequately powered and longer‐term RCTs are required to assess the impact of these agents on hard clinical endpoints such as cardiovascular events, mortality, heart failure and progression of renal disease in T1D patients. We strongly advocate for dedicated outcome trials specifically tailored to T1D to evaluate the efficacy of adjunctive therapies. While extrapolating data from type 2 diabetes (T2D) studies is a logical interim approach, appropriately powered studies in individuals with T1D are needed to fully understand the effects of adjunctive therapies on macrovascular and microvascular complications in this population. Also, further work is required to clarify the subgroup of patients with T1D who would benefit the most from adjunctive therapies.

Attention should also focus on avoiding treatment‐induced complications. The increased risk of DKA associated with SGLT2i use necessitates further investigation into risk mitigation strategies. Future research should aim to identify patient subgroups that are most likely to benefit from SGLT2i while experiencing the least risk of adverse events.

Another critical area for future research is the interaction between adjunctive therapies and advanced diabetes technologies such as CGM and closed‐loop insulin delivery systems. Understanding how these therapies can be integrated to optimise glycaemic control and reduce the risk of hypoglycaemia and other complications is essential. Finally, more understanding of the length of treatment with these agents is required. For example, can these agents be used to induce weight loss and improve IR, after which the treatment is discontinued with a focus on lifestyle factors? Alternatively, these agents can be used intermittently as long‐term safety is currently unclear.

Table 4 summarises gaps in knowledge with the use of metformin, SGLT2i and GLP‐1RA in T1D.

**TABLE 4:** A summary of gaps in knowledge with the use of metformin, sodium‐glucose transporter‐2 inhibitors (SGLT2i) and glucagon‐like peptide receptor agonists (GLP‐1RA in type 1 diabetes (T1D).

Topic	Gaps in knowledge	Future research directions
Hard outcomes and health economic analysis	Limited data on hard outcomes	Conduct adequately powered RCTs to evaluate the impact of these agents on cardiovascular events such as major adverse cardiovascular event (MACE), heart failure hospitalisation, and cardiovascular mortality, and renal disease. Also to explore microvascular complications such retinopathy, nephropathy, and neuropathy outcomes. Health economic analysis should follow the above.
Identification of patient groups	Uncertainty about which patient groups would benefit most from adjunctive treatments	Research to identify patient subgroups that would derive the greatest benefit from these therapies, minimising risks and maximising outcomes (using surrogate outcome measures)
Risk of DKA with SGLT2i	Increased risk of diabetic ketoacidosis (DKA) associated with SGLT2i use highlights a need for an effective risk mitigation strategy	Develop methods to identify patients most at risk and devise management strategies to mitigate DKA risk
Integration with advanced technologies	Lack of comprehensive data on how adjunctive therapies interact with advanced diabetes technologies	Study the integration of these pharmacological agents with technological interventions
Treatment duration and modality	Unclear optimal length and modality of treatment regarding intermittent use versus continuous therapy due to concerns over long‐term safety	Examine the efficacy, safety and health economic impact of using these agents intermittently

Abbreviations: DKA, diabetic ketoacidosis; RCTs, randomised controlled trials.

## 10 CONCLUSION

The management of T1D has significantly evolved over the past decades, yet the quest for optimal glycaemic control and the prevention of complications remains a formidable challenge. This review has highlighted the potential benefits of incorporating adjunctive therapies with cardiovascular protective properties into the treatment regimen for T1D.

While indirect evidence supports the potential benefits of these adjunctive therapies in T1D, hard evidence is lacking, explaining the limited enthusiasm for using these agents in routine clinical practice. Long‐term studies will be crucial to fully elucidate the role of these therapies in T1D management, ensuring their safe and effective use in this population. Moreover, the integration of these therapies with advanced diabetes technologies and the potential for personalised treatment approaches are areas that warrant further investigations.

By addressing both glycaemic control and the multifaceted complications associated with T1D, these therapies offer a comprehensive approach to diabetes care. As we advance in our understanding and management of T1D, these adjunctive therapies could become integral components of personalised diabetes care, enhancing the quality of life and long‐term health outcomes for individuals with T1D.

## CONFLICT OF INTEREST STATEMENT

Ramzi Ajjan: Research grants, honoraria, education support or consulting fees from the Abbott Diabetes Care, AstraZeneca, Bayer, Boehringer Ingelheim, Bristol‐Myers Squibb, Eli Lilly, GlaxoSmithKline, Menarini Pharmaceuticals, Merck Sharp & Dohme and Novo Nordisk. Ahmad Rajab and Sam Pearson No potential conflict of interest relevant to this article was reported.

### PEER REVIEW

The peer review history for this article is available at https://www.webofscience.com/api/gateway/wos/peer-review/10.1111/dom.16332.

## Data Availability

Data sharing not applicable ‐ no new data generated, or the article describes entirely theoretical research.
